# Insight into the phylogeny and antibiotic resistance of *Pseudomonas* spp. originating from soil of the Białowieża National Park in Northeastern Poland

**DOI:** 10.3389/fmicb.2025.1454510

**Published:** 2025-01-22

**Authors:** Wioleta Lewandowska, Jacques Mahillon, Justyna Małgorzata Drewnowska, Izabela Swiecicka

**Affiliations:** ^1^Doctoral School, University of Białystok, Białystok, Poland; ^2^Department of Microbiology and Biotechnology, Faculty of Biology, University of Białystok, Białystok, Poland; ^3^Laboratory of Food and Environmental Microbiology, Earth and Life Institute, Université Catholique de Louvain, Louvain-la-Neuve, Belgium; ^4^Laboratory of Applied Microbiology, Faculty of Biology, University of Białystok, Białystok, Poland

**Keywords:** *Pseudomonas* spp., environmental isolates, Białowieża National Park, phylogeny, antibiotic resistance, soil

## Abstract

The *Pseudomonas* genus includes species present in various environments and known for antibiotic resistance. However, only hospital-associated *Pseudomonas aeruginosa* have been extensively studied regarding antibiotic resistance. Thus, to fill the gap in knowledge on antibiotic resistance among other *Pseudomonas* spp., we investigated 41 isolates from soil samples taken in the Białowieża National Park in Northeastern Poland. This unique forest without notable anthropogenic influence, provides excellent conditions for research of antibiotic resistance from the perspective of natural environments. The phylogeny trees obtained based on the nucleotide sequence of the 16S rRNA gene and *gyrB* gene grouped the isolates into clusters belonging to the *Pseudomonas fluorescens*, *Pseudomonas koreensis*, and *Pseudomonas putida* groups, originating from the *P. fluorescens* lineage. All isolates under study demonstrated resistance to at least 12 out of the 24 antibiotics tested. Resistance to colistin, cefotaxime, and imipenem was detected in 73, 73, and 17% of the isolates, respectively. Most isolates showing resistance to imipenem and colistin clustered within the *P*. *fluorescens* group. Seven isolates were highly multi-resistant, to up to 18 of the 24 antibiotics tested. The presence of resistance genes related to intrinsic resistance of *P. aeruginosa* has been confirmed in environmental isolates.

## Introduction

Since the penicillin discovery by Alexander Fleming in 1928, antibiotics have changed the course of human and animal medicine, specifically in how infectious diseases are treated. However, at the same time, antibiotic resistance has developed in many bacteria and resulted in one of the most critical issues of microbiology nowadays (von Wintersdorff et al., 2016). In particular, a worrisome increase in antibiotic resistance has recently been observed among *Pseudomonas* spp. strains (Behzadi et al., 2021; Botelho et al., 2019; Pang et al., 2019). Moreover, among these bacteria, strains with natural resistance to antibiotics, e.g., aminoglycosides, quinolones, and ß-lactams, have been noted (Behzadi et al., 2021; Azam and Khan, 2019).

*Pseudomonas* spp. are found in a variety of high and low-nutrient ecological niches, including soil and water sites (Govender et al., 2021), as well as in human-related environments (Saati-Santamaría et al., 2022), particularly in hospitals (Vijay et al., 2018). Two of the most common species are *Pseudomonas aeruginosa*, mostly causing nosocomial infections (Rehman et al., 2019) and ventilator-associated pneumonia (Vijay et al., 2018), and *Pseudomonas fluorescens*, found in many clinical samples linked to blood infections (Scales et al., 2014; Silverio et al., 2022). Representatives of these two species are also present in natural environments (Govender et al., 2021; Scales et al., 2014; Silby et al., 2011) due to their broad spectrum of metabolic and environmental adaptation (Silby et al., 2011). Similarly, other *Pseudomonas* species, such as *Pseudomonas putida*, *Pseudomonas syringae*, or *Pseudomonas stutzeri*, on one hand in natural environments can affect functionality through beneficial associations with plants and soil bioremediation (Philippot et al., 2013), but on the other hand can impact human health (Azam and Khan, 2019). Nevertheless, in general, *Pseudomonas* spp. are considered opportunistic pathogens, capable of causing serious health risks, especially to immunocompromised individuals (Silverio et al., 2022).

The main mechanisms that allow *Pseudomonas* spp. to resist antibiotics, include low outer membrane permeability, expression of efflux pumps that pump antibiotics out of the cell, and the production of antibiotic-inactivating enzymes, such as ß-lactamases, that break down or modify antibiotics (Breidenstein et al., 2011). Importantly, antibiotic resistance genetic determinants can be acquired via horizontal gene transfer (HGT), which is often noted among *Pseudomonas* spp. and significantly increases their spread in various environments (Perry and Wright, 2013; Munita and Arias, 2016).

Most of the research on antibiotic resistance concerns bacteria from hospital environments, while those from natural environments are much less studied. Yet, they are suspected to be important reservoirs of antibiotic resistance determinants that may be transferred to clinical strains by various HGT mechanisms (Peterson and Kaur, 2018). Therefore, in order to get a broader view of the antibiotic resistance phenomenon it is necessary to expand our studies on environmental bacteria. In particular, the possible “interlinkages” to environments with limited anthropogenic activities may provide valuable information on the sources and routes of spread of antibiotic resistant genes across bacterial populations.

The Białowieża National Park is the last European natural deciduous and coniferous forest similar to the one covering the area for the last hundreds of years. The origin of the park dates back to 1921 when the Białowieża reserve was established. Then, it was transformed into a national park in 1934 and reinstated in 1947 as the Białowieża National Park (BNP). UNESCO designated BNP as a World Heritage Site, and it is the most crucial central zone of Białowieża Forest Biosphere Reserve, with strict nature protection and limited human activity (Gutowski and Jaroszewicz, 2004). A wide range of studies, including palynological, archeological, and historical ones, indicate a low anthropogenic influence on BNP when compared to other lowland forests, which allows us to consider BNP a living laboratory for biological and evolutionary sciences (Jaroszewicz et al., 2019). For these reasons, BNP is also a significant sampling point to investigate the presence of antibiotic resistance in natural environments. This study aimed to assess the level of antibiotic resistance among bacteria originating from soil samples taken in BNP, with particular attention brought to *Pseudomonas* spp.

## Materials and methods

### Sample collections

**Figure 1:**
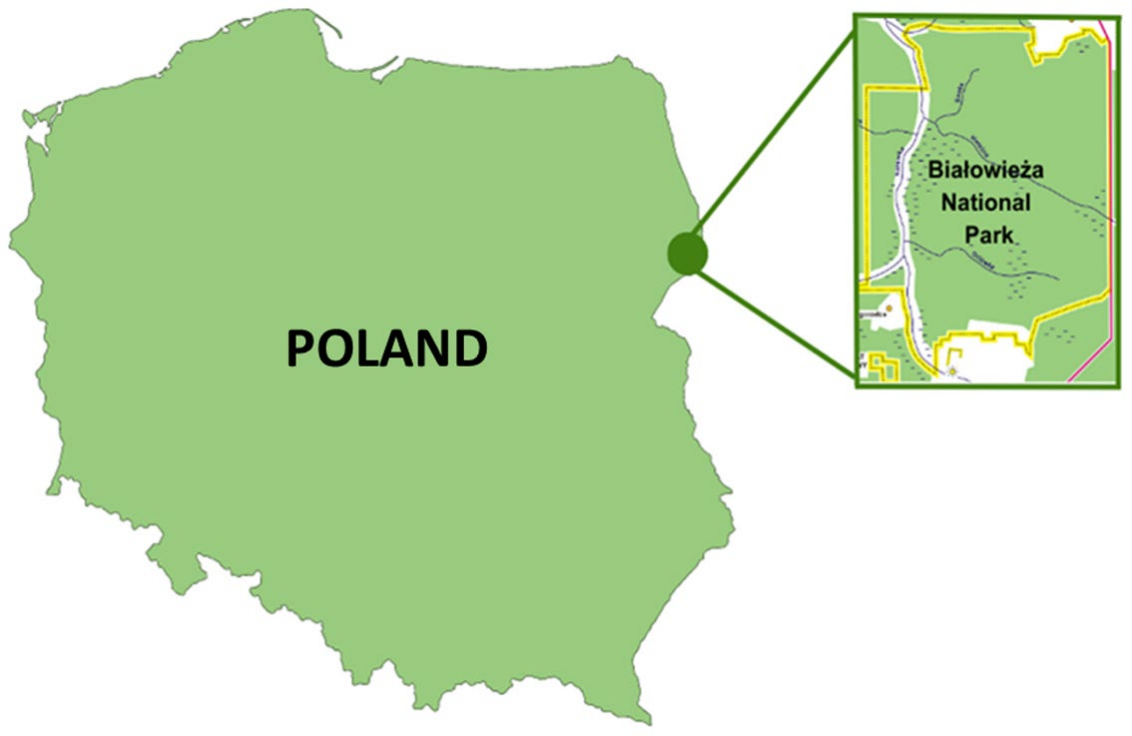
Geographic location of soil sampling at the Białowieża National Park (BNP; N 52° 42′, E 23° 54′) in Northeastern Poland.

Soil samples were collected in May 2019 from the Białowieża National Park (BPN) in Northeastern Poland. Sampling was done strictly following the rules of the Nature Conservation Act adopted on April 16th, 2004, by Polish Parliament (Parliament Diary 2004, No. 92:880) and with the consent of the Ministry of Environment from April 1st, 2019 (Decision No. DOP-WPN.436.48.2019.RS). Altogether, 40 soil samples were taken in triplicate in May 2019 from various forest habitats (Figure 1) distributed over an area of about 50,000 m^2^. Based on the habitat map of the Białowieża National Park, the study plots were selected from (i) coniferous (*n* = 5), (ii) mixed coniferous (*n* = 6), (iii) broadleaved (*n* = 13), (iv) mixed broadleaved (*n* = 9), and (v) alder (*n* = 7). Soil samples were taken aseptically from a depth of *ca.* 10 cm after gently removing the surface soil layer with sterilized laboratory spoons, immediately transported to the laboratory, and stored at −4°C for further analysis. The collection of all samples took place over three following weeks, on May 7th, 14th, and 21st. From 40 soil samples, 360 bacterial isolates were obtained.

### Isolation of antibiotic resistant bacteria

One gram of each soil sample was suspended in 10 mL liquid Luria-Broth (LB) medium containing one of the antibiotics specified in Supplementary Table S1 (Part I) and incubated at 28°C for 72 h with gentle shaking (80–100 rpm). Subsequently, serial 10-fold dilutions (10^−1^ to 10^−5^) of the cultures with sterile phosphate buffered saline were prepared, and 100 μL of the culture suspension from each dilution was plated onto Mueller-Hinton agar with the appropriate antibiotic at the concentration provided in Supplementary Table S1 (Part I). Bacterial cultures onto Mueller-Hinton agar were incubated at 28°C for 96 h. The isolates were further cultured on nutrient agar (A&A Biotechnology, Gdynia, Poland) and characterized by Gram staining. Individual colonies were purified and stored in LB with glycerol (1:1) at −80°C.

### Bacteria classification based on the 16S rRNA gene and the gyrB gene sequences

Bacterial DNA was isolated from 3 mL Luria Broth (LB) overnight cultures incubated at 28°C with the use of the DNeasy Blood & Tissue Kit (Qiagen GmbH, Hilden, Germany) following the manufacturer guideline. An incubation with 20 mg/mL lysozyme for 1 h at 37°C was included to improve cell lysis. The DNA quality was determined using a NanoDrop 2000 Spectrophotometer (ThermoFisher Scientific, Wilmington, United States) and inspected visually on agarose gel (Prona, HISPANAGAR, Burgos, Spain) after electrophoresis.

The primer pairs for the 16S rRNA and *gyrB* (Frank et al., 2008; Baker et al., 2003) genes amplification are presented in Supplementary Table S2. PCR was performed in a SureCycler 8800 (Agilent Technologies) in a final volume of 10 μL and included 5 μL of Qiagen Multiplex PCR Master Mix (Qiagen GmbH, Hilden, Germany), 2 μL of RNase-free water, 1 μL of template DNA and 1 μL of the forward and reverse primers. The amplification protocol included 15 min at 95°C, followed by 30 cycles of 30 s at 94°C, 90 s at 57°C (90 s at 65°C for *gyrB* gene), 90 s at 72°C, and a final extension of 10 min at 72°C. PCR products were purified enzymatically using the EPPiC Fast reagent (A&A Biotechnology). The amplified fragments of the genes were used as templates for DNA sequencing with the ABI3500 automated sequencer (Applied Biosystems) using a BigDye Terminator v3.1 Cycle Sequencing Kit (Applied Biosystems) and an ExTerminator kit (A&A Biotechnology), following the manufacturer guidelines. The nucleotide sequences were assembled with the BioEdit Sequence Alignment Editor v7.2.6.1 and similarity searches were performed using the BLAST (Basic Local Alignment Search Tool) algorithm provided by the National Center for Biotechnology Information (NCBI).^1^ Sequence alignments were prepared using ClustalX 2.1 (Thompson et al., 1994). All obtained sequences were deposited in the National Center for Biotechnology Information (NCBI) GenBank database (for details, see Supplementary Table S4).

### Biochemical activity

Biochemical activities of the *Pseudomonas* spp. isolates were using the API 20 NE kit (bioMérieux, Marcy-l’Étoile, France) according to the manufacturer protocol based on current microbiology standards. After incubation at 28°C for 24 and 48 h, the biochemical reactions and bacterial growth were analyzed according to the manual to determine the numerical profile, allowing for identifying the metabolic patterns of the tested isolates (Geiss et al., 1985). Moreover, the isolates were then tested for cytochrome c oxidase production using Oxidase Reagent (bioMérieux).

### Phylogenetic analysis

Construction of the dendrogram reflecting the phylogenetic relationship of the *Pseudomonas* spp. isolates was achieved using the MEGA11 software (Kumar et al., 2018), applying the Neighbor-Joining algorithm (Saitou and Nei, 1987), and 1,000 bootstrap replicates (Felsenstein, 1985). *Azotobacter vinelandii* NBRC 102612, *P. aeruginosa* ATCC 10145, *Pseudomonas alkylphenolica* JCM 16553, *Pseudomonas brenneri* DSM 15294, *P. fluorescens* ATCC 13525, *Pseudomonas gessardii* DSM 17152, *Pseudomonas helmanticensis* DSM 28442, *Pseudomonas huaxiensis* JCM 32907 *Pseudomonas laurentiana* JCM 32154, *P. putida* ATCC 12633, *Pseudomonas trivialis* DSM 14937 T and *Pseudomonas yamanorum* DSM 26522 strains were used as references in the phylogenetic tree based on the 16S rRNA gene sequences. *A. vinelandii, P. aeruginosa* PAO1*, P. fluorescens, P. gessardii, Pseudomonas koreensis, P. putida, P. trivialis*, and *P. yamanorum* strains were used as references in the phylogenetic tree based on the *gyrB* gene sequences.

### Antibiotic susceptibility profiling of *Pseudomonas* spp. isolates

Antimicrobial resistance patterns were determined by the MIC Test Strip (Liofilchem MTS, Italy) gradient diffusion method achieved on Mueller–Hinton agar, against 24 antibiotics (Supplementary Table S1, Part II), following manufacturer guidelines. After 24 h of incubation at 28°C, bacteria were classified as sensitive, intermediate or resistant to the antibiotics used according to the Clinical Laboratory Standards Institute Guidelines (CLSI, 2021) and the European Committee on Antimicrobial Susceptibility Testing guidelines (EUCAST, 2021a,b). *P. aeruginosa* reference strains ATCC 19582 and ATCC 27853 were included as quality control. As proposed by Govender et al. (2021), the isolates demonstrating resistance to at least three antibiotics were considered multidrug resistant. The multi-antibiotic resistance index (a MAR index) was calculated according to Equation 1 (Blasco et al., 2008):


(1) MAR=a/b

where “*a*” means the number of antibiotics the isolate was resistant to, while “*b*” indicates the total number of antibiotics against which the isolate was tested.

The MAR index value greater than 0.2 reflects a high-risk area with possible exposure to antibiotics, while MAR index equal to or lower than 0.2 indicates bacteria without previous exposure to such antibiotics (Odjadjare et al., 2012).

### Detection of genes associated with antimicrobial resistance

The antibiotic resistance genes were detected with PCR using primers developed for each gene with the Geneious Prime program (Supplementary Table S2). The primers were designed based on the gene sequences of the reference strains available in the NCBI database (Supplementary Table S3). The PCRs were performed in a SureCycler 8800 (Agilent Technologies) in a final volume of 15 μL and included 7.5 μL of StartWarm PCR Mix (A&A Biotechnology), 4 μL of RNase-free water, 1.5 μL of template DNA and 1 μL of the forward and reverse primers. The amplification consisted of 5 min at 95°C, followed by 30 cycles of 30 s at 94°C, 30 s at depending on the primer pair (Supplementary Table S2), 45 s at 72°C, and a final extension of 5 min at 72°C. The amplified products were inspected visually by agarose gel (Prona) electrophoresis and staining with Midori Green (NIPPON Genetics Europe, Germany).

## Results and discussion

### Phylogeny portrait of *Pseudomonas* spp. from the Białowieża National Park

**Figure 2:**
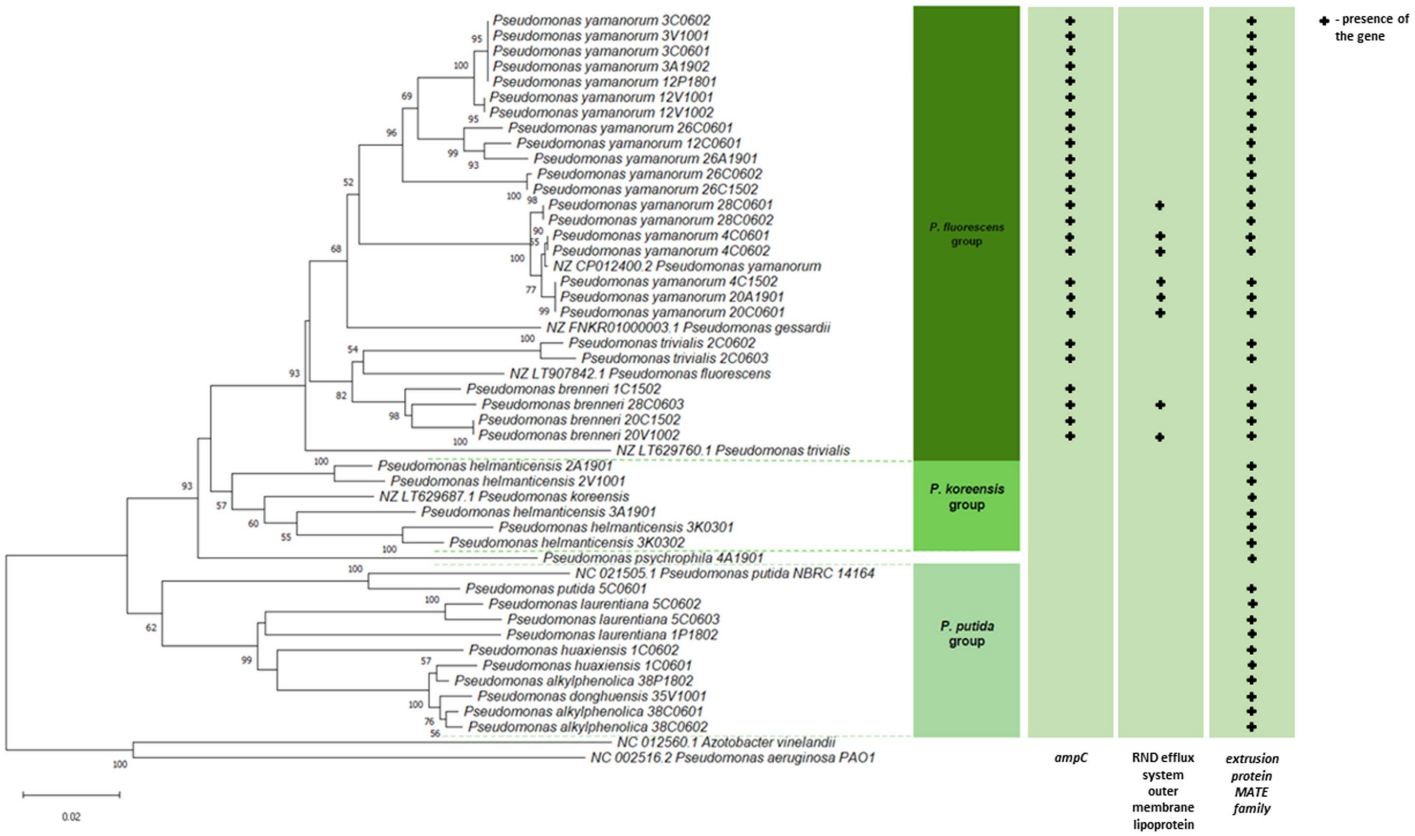
Phylogenetic relatedness of *Pseudomonas* spp. isolated from soil samples taken in the Białowieża National Park (BNP). The phylogenetic tree of the 41 isolates and 8 reference strains (in green) was constructed based on partial *gyrB* sequences (820 nt). The evolutionary history was inferred using the Neighbor-Joining (NJ) method implemented in the MEGA11 software (Kumar et al., 2018). Branch quality was evaluated using a 1,000 replicates bootstrap test. All bootstrap supporting values of ≥50% in which the associated taxa clustered together are shown next to the branches. The optimal tree is drawn to scale, with branch lengths in the same units as those of the evolutionary distances used to infer the phylogenetic tree.

Natural dynamics have strongly shaped BNP forest structure, composition, and function without notable anthropogenic influence over a long period. This area is unique in Europe, with continuous forests overlaying the same area for nearly 12,000 years (Jaroszewicz et al., 2019). The composition and activity of bacterial communities follow seasonal patterns but are also shaped by short-term factors such as precipitation, snowmelt, drought (López-Mandéjar et al., 2015). The spatial and temporal heterogeneity of the soil bacterial community, combined with the complexity of the forest habitat, make their comprehensive investigation highly challenging (Lladó et al., 2018). Sampling soil in May 2019 focuses our attention on a specific temporal snapshot of soil microbiota functioning. Nevertheless, antibiotic resistance present in the environment changes but does not diminish over time. It is influenced not only by the presence of antibiotic resistant microorganisms but also by the pool of genes and extracellular DNA fragments existing within a given ecosystem. It is important that seasonal variation is a latent variable, which is related to shifting biotic and abiotic factors resulting from anthropogenic impacts. Seasonal variability plays an important role in shaping soil antibiotic resistance profiles (Xiang et al., 2021). The majority of such studies, however, focus on environments altered by human activity, where the impact of seasonality on microbial communities is most pronounced. Our aim was to conduct a preliminary screening of resistant *Pseudomonas* strains present in the soil of the BPN unaffected by anthropogenic influence. In our study, we tested *Pseudomonas* spp. obtained from the soil samples from BNP, taking into account the identification and the confirmation of the presence of colistin-resistant and carbapenem-resistant strains (Figure 1). The isolates were identified at the genus level based on the type of growth, microscopic observations, and API 20NE tests (Supplementary Table S4). Considering the forest habitats, eight isolates were obtained from soil samples taken in the forest with alder as the dominant tree, 24 isolates from the broadleaved soil samples, and nine isolates from the mixed broadleaved soil samples (for details, see Supplementary Table S4). Based on the nucleotide sequence of the 16S rRNA gene fragment (1,318 nt) of the isolates under study and the 16S rRNA sequences of reference *Pseudomonas* spp. strains from the NCBI database, a phylogenetic tree was constructed showing the degree of relatedness between individual BNP isolates (Supplementary Figure S1). *Pseudomonas* spp. are very difficult to discriminate so, to confirm the affiliation at the species level, the additional genetic tree was created based on the housekeeping gene *gyrB* nucleotide sequence and the corresponding sequences of reference *Pseudomonas* spp. strains from the NCBI database (820 nt) (Figure 2). The use of 16S rRNA sequences allows the assignment of strains to genus and confirmation of phylogenetic relationships. However, the accuracy of the 16S rRNA gene sequence at the intrageneric level is low, especially in the case of very closely related strains such as *Pseudomonas* spp. In that case the “housekeeping” gene sequences may provide better resolution thus we decided to focus on the *gyrB* gene encodes the beta-subunit of gyrase. In combination with the 16S rRNA gene sequence has proven reliable for species differentiation and strain identification (Gomila et al., 2015).

As shown in Figure 2, the bootstrap values and genetic distances identified four phylogenetic clusters, all belonging to the *P. fluorescens* lineage (Peix et al., 2018; Sawada et al., 2020). Cluster I covered the highest number of bacteria (*n* = 25) of the *P. fluorescens* group (Peix et al., 2018), where representatives of the *P. yamanorum*, *P. brenneri*, *P. trivialis* were present. Cluster II was represented by one isolate *Pseudomonas psychrophila*. Cluster III (*n* = 5) comprised the only representative of *P. helmanticensis*, that belong to the *P. koreensis* group (Lauritsen et al., 2021), while the last cluster IV (*n* = 10) included *P. alkylphenolica*, *Pseudomonas donghuensis, P. huaxiensis*, *P. laurentiana*, and *P. putida* from the *P. putida* group (Peix et al., 2018). A phylogenetic tree based on the *gyrB* housekeeping gene sequences confirmed species affiliation and belonging of the tested isolates to four phylogenetic clusters (Figure 2). The composition and the location of clusters unreservedly correspond to those from the phylogenetic tree constructed based on the 16S rDNA sequences. The construction of two phylogenetic trees using two different genes allowed for the precise determination of the taxonomic identity of the strains into species (Figure 2; Supplementary Figure S1).

Interestingly, the most abundant species in BNP, *P. yamanorum* and *P. brenneri*, are both psychrotolerant bacteria (Arnau et al., 2015; van den Beld et al., 2016), and *P. helmanticensis* is also considered psychrophilic (Kumar et al., 2020). This is in line with our previous long-term studies of the BNP microorganisms, where most bacteria are well adapted to low temperatures (Drewnowska and Swiecicka, 2013; Fiedoruk et al., 2021).

In general, members of the *Pseudomonas* genus show high metabolic and physiologic versatility, also noted in our study, enabling them to colonize diverse habitats (Igbinosa et al., 2012). In soil, these bacteria decompose dead plant biomass and fungal mycelia, interact with plant roots and mycorrhizal fungi as commensals or mycorrhiza helpers (Lladó et al., 2017), or contribute to plant health by either antagonizing plant-pathogenic microorganisms (Fischer et al., 2009) or through direct influence on the plant growth (Molina et al., 2020). Such functions are possible thanks to their diverse metabolic activities, especially in the cycling of carbon, nitrogen, and phosphorus. In our study, all *Pseudomonas* spp. isolates assimilated glucose, potassium gluconate, capric acid, malate, and sodium citrate, but none could synthesize indole, ferment glucose or assimilate maltose. The 4A1901 isolate was capable of producing urease, while 38C0601 hydrolysed esculin. More metabolic diversity was observed among the isolates concerning the reduction of nitrates to nitrites (denitrification process), monosaccharides (arabinose, mannose, N-acetyl-glucosamine) decomposition, or phenyl-acetic acid assimilation, a phytohormone found in plants (Perez et al., 2023). Of note, five isolates (38P1802, 38C0601, 38C0602, and 35V1001), could not produce cytochrome oxidase, a feature characteristic of clinical *Pseudomonas* spp. Interestingly, contrary to the isolates classified into the I-III phylogenetic clusters, isolates of cluster IV (*P. putida*) were unable to assimilate arabinose and mannose.

### Antibiotic resistance profiling of *Pseudomonas* spp. from BNP

**Figure 3:**
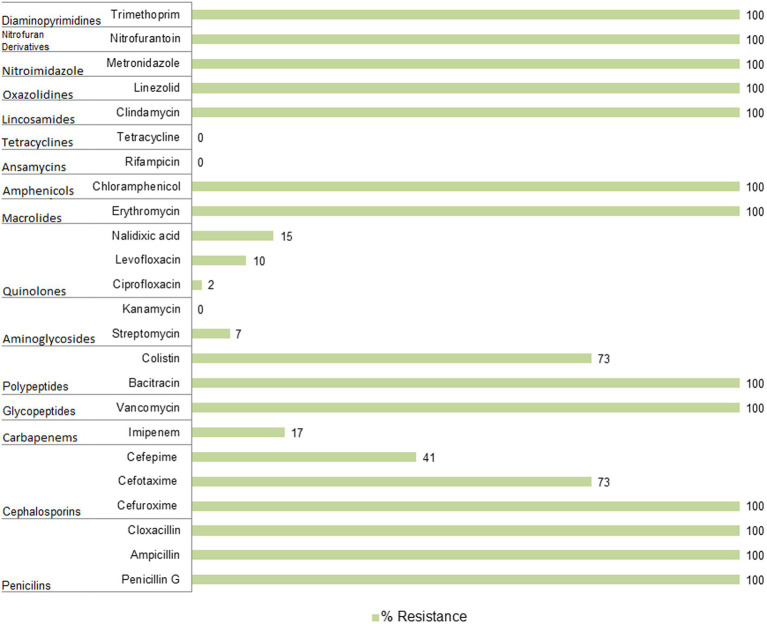
Antibiotic resistance of *Pseudomonas* spp. isolated from soil samples collected in the Białowieża National Park (BNP).

*Pseudomonas* spp. resistance has mainly been examined concerning β-lactams, quinolones, and sulfonamides (Devarajan et al., 2017; Kittinger et al., 2016; Spindler et al., 2012). However, we applied a much wider spectrum of antibiotics to assess more complex resistance profiles and to confirm whether the same pattern of intrinsic resistance occurs in environmental strains. Altogether, we tested 24 antibiotics representing 15 antibiotic families or subfamilies being commonly used in human and/or veterinary medicine (Figure 3; Supplementary Table S5). Resistance to ampicillin, bacitracin, cefuroxime, chloramphenicol, clindamycin, cloxacillin, erythromycin, linezolid, metronidazole, nitrofurantoin, penicillin G, trimethoprim and vancomycin was common in all isolates under study. In addition, a high number of the BNP *Pseudomonas* spp. showed resistance to cefotaxime (*n* = 30, 73%), colistin (*n* = 30, 73%), cefepime (*n* = 17, 41%), imipenem (*n* = 7, 17%) and nalidixic acid (*n* = 6, 15%). All isolates were susceptible to kanamycin, rifampicin, and tetracycline, while less than 15% displayed resistance to ciprofloxacin, levofloxacin, or streptomycin.

Resistance of the BNP *Pseudomonas* spp. to imipenem, belonging to the carbapenem family, is particularly noteworthy as this antibiotic is an essential last line β-lactams used to treat multidrug resistant infections (Zhuang et al., 2021). Carbapenem resistant *P. aeruginosa* isolates have been described as priority pathogens that threaten human health (WHO, 2017). Seven isolates from this study (17%) showed resistance to imipenem. Interestingly, most of them clustered together within the *P. fluorescens* group (Figure 2; Supplementary Table S5), which may suggest a common evolutionary history of imipenem resistance among these environmental isolates. This also indicates that carbapenem-resistant *Pseudomonas* spp. are present in BNP, an environment with low to no human activity. Interestingly, resistance to imipenem among *Pseudomonas* spp. has been noted in other studies with lower frequencies: 7% for bacteria from wastewater and surface water (Govender et al., 2021), 2.1% for isolates from the Danube River (Kittinger et al., 2016) or 1.9% for isolates obtained from fecal wastes in the environment and contaminated surface water (Camiade et al., 2020).

Similarly to imipenem, colistin (polymyxin E) is also used in human medicine, especially as the last line for controlling carbapenem resistant *Enterobacteriaceae* (Eichenberger and Thaden, 2019). Moreover, this antibiotic has a long history of prophylactic use in animal production. Some countries have banned its use in farming to limit its influence on antibiotic resistance (Zhuang et al., 2021). High frequency of colistin-resistance among the BPN *Pseudomonas* spp. (73%), mainly clustered within the *P. fluorescens* group (Figure 2; Supplementary Table S5), is a cause for concern. Camiade et al. (2020) found much lower (0.6%) resistance to colistin, as did Govender et al. (2021) (7%). The BNP *Pseudomonas* spp. resistances to ciprofloxacin, levofloxacin and streptomycin were similar to those noted in other studies (Govender et al., 2021; Camiade et al., 2020; Kittinger et al., 2016).

**Figure 4:**
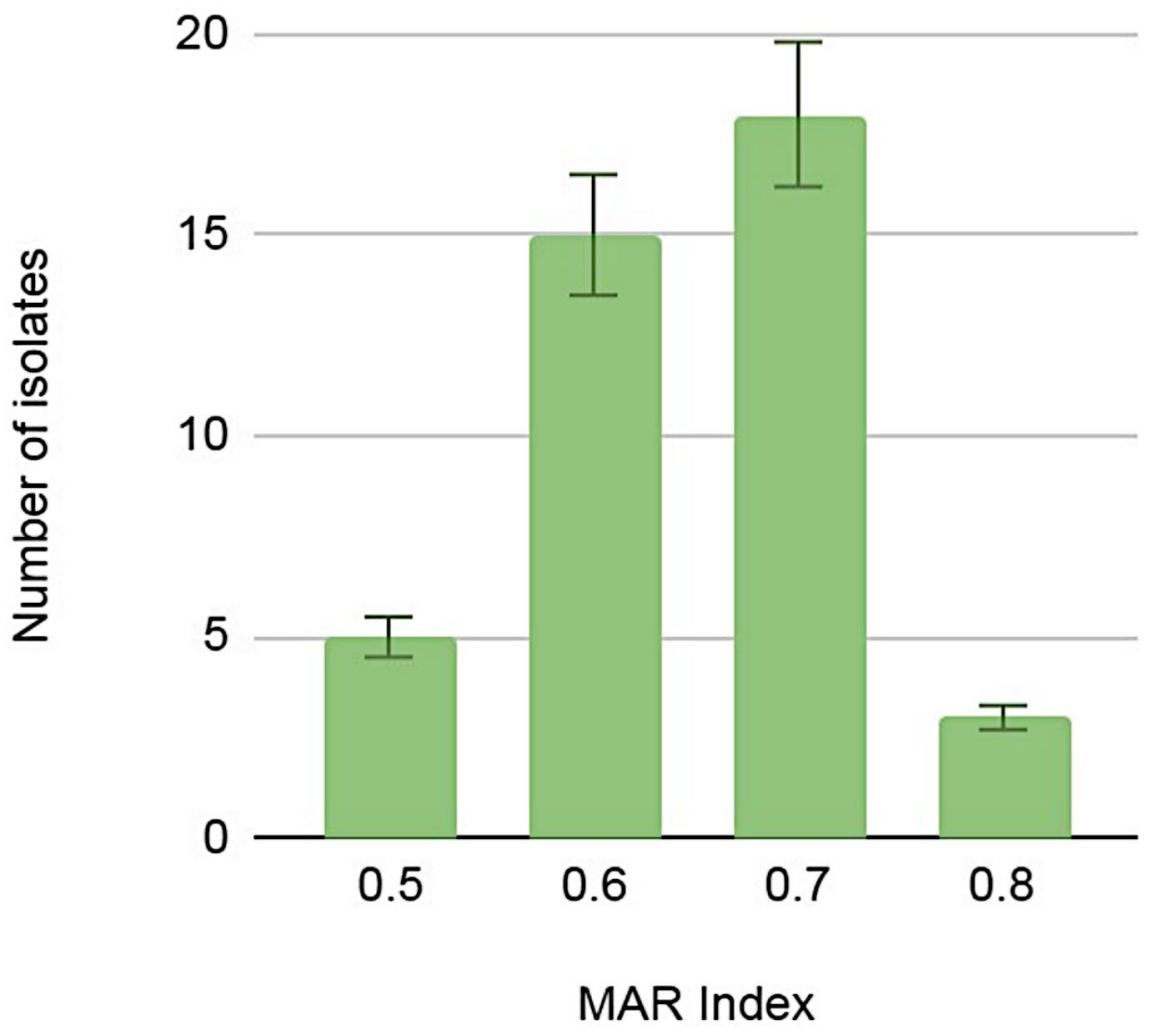
Numbers of *Pseudomonas* spp. isolates demonstrating multi-antibiotic resistance index (MAR) calculated as a quotient of the number of antibiotics to which the isolate was resistant and the total number of antibiotics against which the isolate was tested.

The level of multi-resistance varied significantly from one isolate to another, demonstrating heterogeneous antibiotic resistance signatures within the *Pseudomonas* genus. Nevertheless, we noted an unprecedented level of multi-resistance in bacteria occupying the BPN natural environment that has limited anthropogenic influence and potentially no antibiotic contamination. Seven isolates displayed resistance to up to 18 antibiotics used in the screening. The multiple antibiotics resistance (MAR) index is widely used to evaluate antibiotic resistance and its associated health risks (Albini et al., 2022; Kurdi Al-Dulaimi et al., 2019). The BNP *Pseudomonas* spp. isolates showed a significant variation in the index, ranging from 0.5 to 0.8 (Figure 4; Supplementary Table S5), but most of the isolates (*n* = 18) indicated the MAR index at 0.7.

### Presence of genes associated with antimicrobial resistance

The presence of three genes involved in the *Pseudomonas* spp. antibiotic resistance was further investigated. Since *P. aeruginosa* intrinsically possesses class C AmpC enzymes, chromosomally encoded cephalosporinases (Eichenberger and Thaden, 2019), we tested the occurrence of the *ampC* gene in the environmental *Pseudomonas* spp. under study, and confirmed its presence in 61% (*n* = 25) of tested isolates. They were all resistant to at least one of the three cephalosporin antibiotics tested and clustered within the *P. fluorescens* group on the phylogenetic tree (Figure 2; Supplementary Table S5).

The outer membrane efflux lipoprotein from the NodT family of the RND (Resistance-Nodulation-Cell-Division) type efflux system plays a key role in antibiotic resistance in *P*. *aeruginosa* (Li et al., 2015; Pang et al., 2019). We tested the occurrence of the lipoprotein *nodT* and confirmed its presence in nine of them (e.g., 4C1502, 20A1901, 4C0601, 4C0602, 28C0601, 28C0602, 20C0601, 20V1002, and 28C0603), also all clustered within the *P. fluorescens* group (Figure 2; Supplementary Table S5).

Antimicrobial extrusion protein Na+/drug antiporter belonging to the MATE (multidrug and toxic compound extrusion) family of efflux pumps, recognizes compounds with different chemical structures as substrates and exports them using the electrochemical gradient of Na+ as the driving force. MATE may be associated with multidrug resistance (Kusakizako et al., 2020). The presence of the gene coding MATE was confirmed in all tested isolates (Supplementary Table S5).

## Conclusion

This study provides insights into the importance of soil in the distribution of antibiotic resistant bacteria and adds further weight to growing concerns about the relevance of natural environmental antibiotic resistance. *Pseudomonas* spp. from the Białowieża National Park (BNP) was shown to be resistant to ampicillin, bacitracin, cefuroxime, chloramphenicol, clindamycin, cloxacillin, erythromycin, linezolid, metronidazole, nitrofurantoin, penicillin G, trimethoprim, and vancomycin (resistance demonstrated by all isolates under study), but also to cefotaxime, colistin, cefepime, imipenem, and nalidixic acid (resistance observed in 6–73% of the isolates). While all isolates were susceptible to kanamycin, rifampicin, and tetracycline. Moreover, in environmental isolates we observed the presence of an intrinsic resistance pattern of *P. aeruginosa* associated with hospital niches. In this context, we need additional research on factors correlated with the occurrence of antibiotic resistance phenomenon, as well as ecological conditions that could notably affect antibiotic resistance.

## Data Availability

The datasets presented in this study can be found in online repositories. The names of the repository/repositories and accession number(s) can be found in the article/Supplementary material.

## Footnotes

^1^ http://blast.ncbi.nlm.nih.gov/Blast.cgi
